# Spectroscopic imaging of D-2-hydroxyglutarate and other metabolites in pre-surgical patients with IDH-mutant lower-grade gliomas

**DOI:** 10.1007/s11060-022-04042-3

**Published:** 2022-06-08

**Authors:** Adam W. Autry, Marisa Lafontaine, Llewellyn Jalbert, Elizabeth Phillips, Joanna J. Phillips, Javier Villanueva-Meyer, Mitchel S. Berger, Susan M. Chang, Yan Li

**Affiliations:** 1 Present Address: Department of Radiology and Biomedical Imaging, University of California San Francisco, San Francisco, CA, USA; 2 Department of Pathology, University of California San Francisco, San Francisco, CA, USA; 3 Department of Neurological Surgery, University of California San Francisco, San Francisco, CA, USA

**Keywords:** Lower-grade glioma, D-2-Hydroxyglutarate, IDH, MRSI, Image-guided

## Abstract

**Purpose:**

Prognostically favorable IDH-mutant gliomas are known to produce oncometabolite D-2-hydroxyglutarate (2HG). In this study, we investigated metabolite-based features of patients with grade 2 and 3 glioma using 2HG-specific in vivo MR spectroscopy, to determine their relationship with image-guided tissue pathology and predictive role in progression-free survival (PFS).

**Methods:**

Forty-five patients received pre-operative MRIs that included 3-D spectroscopy optimized for 2HG detection. Spectral data were reconstructed and quantified to compare metabolite levels according to molecular pathology (IDH1^R132H^, 1p/19q, and p53); glioma grade; histological subtype; and T2 lesion versus normal-appearing white matter (NAWM) ROIs. Levels of 2HG were correlated with other metabolites and pathological parameters (cellularity, MIB-1) from image-guided tissue samples using Pearson’s correlation test. Metabolites predictive of PFS were evaluated with Cox proportional hazards models.

**Results:**

Quantifiable levels of 2HG in 39/42 (93%) IDH+ and 1/3 (33%) IDH− patients indicated a 91.1% apparent detection accuracy. Myo-inositol/total choline (tCho) showed reduced values in astrocytic (1p/19q-wildtype), p53-mutant, and grade 3 (vs. 2) IDH-mutant gliomas (*p* < 0.05), all of which exhibited higher proportions of astrocytomas. Compared to NAWM, T2 lesions displayed elevated 2HG+γ-aminobutyric acid (GABA)/total creatine (tCr) (*p* < 0.001); reduced glutamate/tCr (*p* < 0.001); increased myo-inositol/tCr (*p* < 0.001); and higher tCho/tCr (*p* < 0.001). Levels of 2HG at sampled tissue locations were significantly associated with tCho (R = 0.62; *p* = 0.002), total NAA (R = − 0.61; *p* = 0.002) and cellularity (R = 0.37; *p* = 0.04) but not MIB-1. Increasing levels of 2HG/tCr (*p* = 0.0007, HR 5.594) and thresholding (≥ 0.905, median value; *p* = 0.02) predicted adverse PFS.

**Conclusion:**

In vivo 2HG detection can reasonably be achieved on clinical scanners and increased levels may signal adverse PFS.

## Introduction

Diffuse infiltrating gliomas present as a family of diseases that demonstrate heterogenous malignancy and survival characteristics. Following the discovery that 70–90% of lower-grade (2/3) gliomas (LrGG) [1, 2] carry prognostically-favorable mutations in *isocitrate dehydrogenase 1 & 2* (*IDH 1/2*), molecular classification has become increasingly emphasized under the precision medicine paradigm [1-6]. According to WHO 2021 classification guidelines [7], IDH mutations are now treated as exclusive features of LrGG, with 1p/19q-codeletion being used to further separate oligodendroglial versus astrocytic subtypes. Besides their prognostic significance and biologic implications [8], IDH mutations hold considerable importance based on the resultant enzymatic production of oncometabolite D-2-hydroxyglutarate (2HG) [9], which induces broad metabolic reprogramming [10] and functions as a marker of tumor that can confirm mutational status.

Non-invasive detection of 2HG was developed through a variety of _1_H magnetic resonance spectroscopy (MRS) techniques [11-14]. These include the short echo time (TE = 30 ms) with spectral fitting [13]; long TE (97–110 ms) that exploits the unique signal evolution of 2HG’s strongly coupled spin system [14, 15]; and spectral editing methods (TE = 68 ms) designed to isolate 2HG from background resonances via coupling patterns [11, 14]. Clinical applications of 2HG MRS have shown diagnostic utility in identifying gliomas that express a range of IDH1/2 mutations [16, 17] and reliably quantifying concentrations in excess of 1 mM [18]. Moreover, levels of 2HG quantified from spectral data were demonstrated to increase with glioma progression [18] and decrease in response to treatment [18-20], while also displaying correlations with tumor cellularity [12, 13, 18, 20]. Patient lesions presenting with IDH2, versus IDH1, mutations have notably been characterized spectroscopically by more prodigious formation of 2HG [21, 22].

In this study, we investigated metabolomic characteristics of patients with IDH-mutant LrGG prior to surgery using a 2HG-targeted, long-TE [14] implementation of 3-D MR spectroscopic imaging (MRSI) that enabled whole-brain coverage. The goals were to evaluate metabolite features of molecular-based classifications of LrGG, correlate them with image-guided tissue pathology, and assess their predictive role on progression-free survival (PFS).

## Methods

### Patient population

Forty-five patients diagnosed with new or recurrent LrGG and presenting for surgical resection were recruited to this IRB-approved study following informed consent. A summary of patient population characteristics is provided in Table 1. The median age at recruitment was 34 years, with a range of 19–72 (60% male; 40% female).

### Pre-surgical MRI and MRSI

Pre-surgical MR imaging was acquired using a 3 Tesla scanner (GE Healthcare Technologies, Waukesha, WI) equipped with an 8-channel phased-array headcoil. The anatomical imaging protocol included T2-weighted fluid-attenuated inversion recovery (FLAIR), T1-weighted inversion recovery spoiled gradient echo (IR-SPGR) pre/post-gadolinium images, diffusion-weighted images acquired in the axial plane with six gradient directions (TR/TE = 1000/108 ms, voxel size = 1.7 × 1.7 × 3 mm^3^, b = 1000 s/mM^2^), and perfusion-weighted images acquired using a T2*-weighted echo-planar imaging sequence (TR/TE = 1250–1500/35–54 ms, 30–35° flip angle, 128 × 128 matrix, 3–5 mM slice thickness, 7–15 slices; 3 mL/s bolus injection of 0.1 mmol/kg gadolinium diethyltriamine pentaacetic acid). The 2HG-specific 3-D MRSI data were acquired using point-resolved spectroscopic selection (PRESS) (TR/TE1/TE2 = 1500/32/65 ms, matrix size = 18 × 18 × 16, nominal voxel size = 1 × 1 × 1 cm^3^) and flyback echo-planar readout, with a total acquisition time of 8.2 min [14, 23].

### MRSI post-processing

MRSI data were reconstructed for the full natively acquired 3D volumes, as previously described [24], and for correlative analysis of spectra versus tissue assays, spectral voxels were recentered at the locations of image-guided tissue. The choline-to-*N*-acetylaspartate index (CNI) was computed automatically for each voxel using in-house software [25]. Quantification of metabolite data was performed using LCModel spectral fitting [26], with Cramer-Rao lower bounds (CRLBs) ≤ 10% for total choline (tCho; phosphocholine, glycerophosphocholine, choline), total creatine (tCr: creatine, phosphocreatine), total NAA (tNAA; *N*-acetylaspartate, *N*-acetylaspartylglutamate); 20% for glutamate (Glu), glutamine (Gln), myo-inositol (Ml), and glycine (Gly); and 25% for 2HG and GABA. Metabolite levels were expressed relative to tCr.

### Anatomical segmentation and ROIs

The T2 lesion (T2L) and contrast-enhancing lesion (CEL) were segmented using 3D Slicer [27]. Normal-appearing white matter (NAWM) ROIs were generated by segmenting pre-contrast T1-weighted images using the FSL FAST algorithm [28] and subtracting the T2L. Volumes of each ROI were determined along with the spectroscopically-defined region of CNI_T2L_ > 2 in the T2L. MRSI voxels containing ≥ 50% T2L or ≥ 75% NAWM volumes were classified according to these respective ROIs.

### Image-guided tissue sampling

Based on pre-surgical MRI parameters, suspected tumor within the T2 lesion was designated for intra-operative tissue sampling. Regions with low apparent diffusion coefficients (ADC), high CNI (> 2; metabolic lesion), and/or elevated cerebral blood volume (CBV) were defined as 5-mm spherical targets using BrainLab (BrainLab Inc., Munich, Germany) surgical navigation software [29]. Additionally, targets had been chosen according to surgical accessibility and heterogeneity criteria that stipulated spatially distributed sampling. Surgeons located and removed these image-guided targets during surgery at their discretion, and the LPS-coordinates for the actual site of tissue removal were recorded. Tissue was acquired early in the surgery to minimize potential tissue shift that would impact registration with imaging. Upon excision, samples were immediately fixed in 10% zinc formalin, dehydrated by graded ethanols, and embedded in Paraplast Plus wax (McCormick Scientific LLC, St. Louis, MO) using standardized immunohistochemistry protocols.

### IHC parameters

A single pathologist performed immunohistochemical analysis of the image-guided tissue samples. While CNS WHO grading [7] characterized the overall tumor, each image-guided tissue sample was assessed according to tumor cellularity, measured by the mean number of cells within a 200 × field-of-view (0.776 mM^2^), and proliferation (MIB-1 index), as determined by immunostaining with Ki-67 (clone MIB-1; Ventana, 790-4286). IDH mutation (IDH+) was assessed primarily via immunostaining with an anti-IDH1^R132H^ antibody (Dianova, H09), and in some instances by sequencing targeted to IDH1/2 mutational variants, allowing IDH-wildtype tumor to also be identified. Fluorescence in situ hybridization (FISH) analysis was used to assess 1p/19q co-deletion and immunostaining was performed for p53 mutation. Strong nuclear staining for p53 in ≥ 10% of tumor cells was used as a surrogate for TP53 mutation [30].

### Statistical analysis

Wilcoxon rank sum tests in R were used to compare MRSI and volumetric parameters from IDH+ patients, according to ROIs, glioma grade, histological subtype, and status of pathological mutation (p53). Correlations were performed in R among continuous metabolite and pathology parameters (Pearson), and ordinal pathology parameters (Kendall-Tau). The influence of metabolite parameters on progression-free survival (PFS) was evaluated with Cox proportional hazards models, and PFS was compared between subgroups using the log-rank test. Due to the exploratory nature of this study, correction for multiple comparisons was not made.

## Results

### WHO 2021 tumor characterization

A total of 108 image-guided tissue samples were acquired from 45 patients undergoing tumor resection as part of standard of care at UCSF. Table 1 details pathological and molecular tumor characteristics within the patient population. Analysis of the tissue revealed 42/45(93%) patients with IDH-mutant diffuse glioma, determined on the basis of IHC staining for the IDH1^R132H^ mutation (*n* = 39), *IDH1* sequencing (*n* = 1) and *IDH2* sequencing (*n* = 2). The remaining 3/45(7%) gliomas were verified as IDH1-negative by both IDH1^R132H^ immunostaining and *IDH1* sequencing; *IDH2* sequencing showed no mutation in 2/3 of these cases, with the third case being unanalyzable but unlikely *IDH2* mutated (p53-negative, 1p/19q intact). Among the 42 patients with IDH-mutant tumors (hereafter referred to as IDH+) there were 29(69%) grade 2 gliomas (13 oligodendrogliomas; 16 astrocytomas) and 13(31%) grade 3 gliomas (3 oligodendrogliomas; 10 astrocytomas). A majority of IDH+ patients (37/42; 88%) were newly diagnosed with LrGG and formed the focus of reported findings based on their treatment-naïve status, while some patients (5/42; 12%) had at least one recurrence. Pathology demonstrated p53 expression in 27/34 (79%) assayed newly diagnosed tumors.

With respect to radiological presentation, the IDH+ patients displayed mostly non-enhancing tumors (36/42; 86%) on anatomical MRI, with mean ± standard deviation T2L volumes of 48.3 ± 36.9 cm^3^ and CEL volumes (*n* = 6) ranging 0.14–2.58 cm^3^.

### In vivo *brain metabolism*

**Fig. 1:**
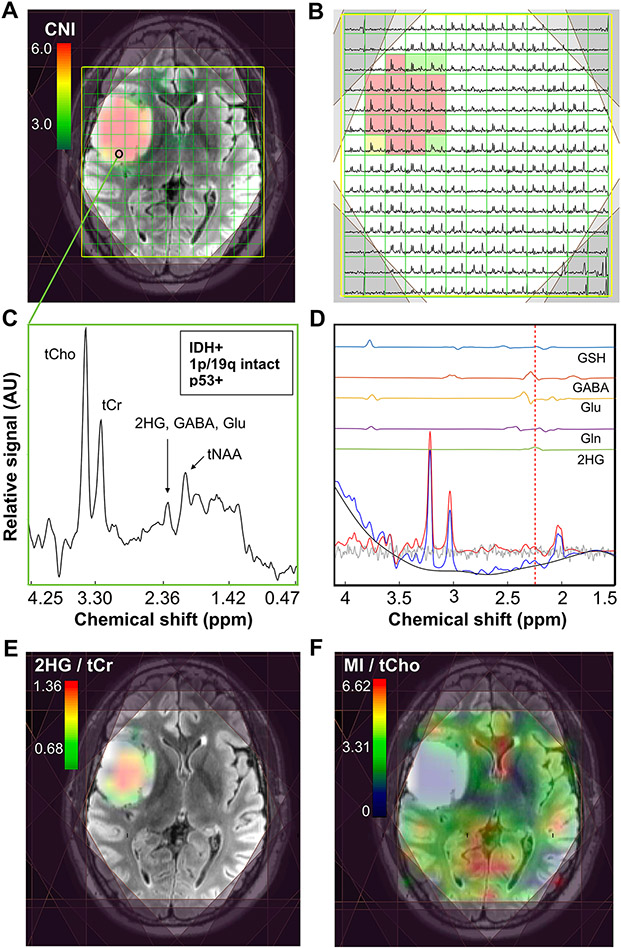
2HG-specific MRSI. Spectral data acquired prior to surgery from a patient with newly diagnosed IDH-mutant grade 2 astrocytoma. A CNI map overlaid on a FLAIR image (**A**) and corresponding spectra (**B**) illustrate elevated levels of choline species associated with cell turnover in the lesion; the black ring in the T2 lesion indicates where tissue was surgically sampled. Spectra reconstructed (**C**) and quantified (**D**) at the site of tissue sampling highlight the 2HG resonance among other common brain metabolites. The map of 2HG/tCr (**E**) shows the localization of 2HG within the T2L, while the map of MI/tCho (**F**) is conversely elevated in normal-appearing tissue

Example pre-surgical MRSI data from a patient presenting with newly diagnosed IDH+ grade 2 astrocytoma are shown in Fig. 1. A CNI map overlaid on a FLAIR image highlights the metabolic abnormality within the T2 lesion (Fig. 1A), as illustrated by the corresponding spectra (Fig. 1B). Data reconstructed at the location of surgical tissue sampling indicated by the black ring show the 2HG resonance along-side other common brain metabolites (Fig. 1C). Based on LCModel quantification, the fitted contributions of individual metabolites were determined in the region of 2HG, with the red-dotted line depicting the specific chemical shift of 2HG at 2.26 ppm (Fig. 1D). The map of 2HG/tCr (Fig. 1E) demonstrates the localization of the oncometabolite within the T2L; whereas MI/tCho (Fig. 1F) increases outside of the lesion.

**Fig. 2:**
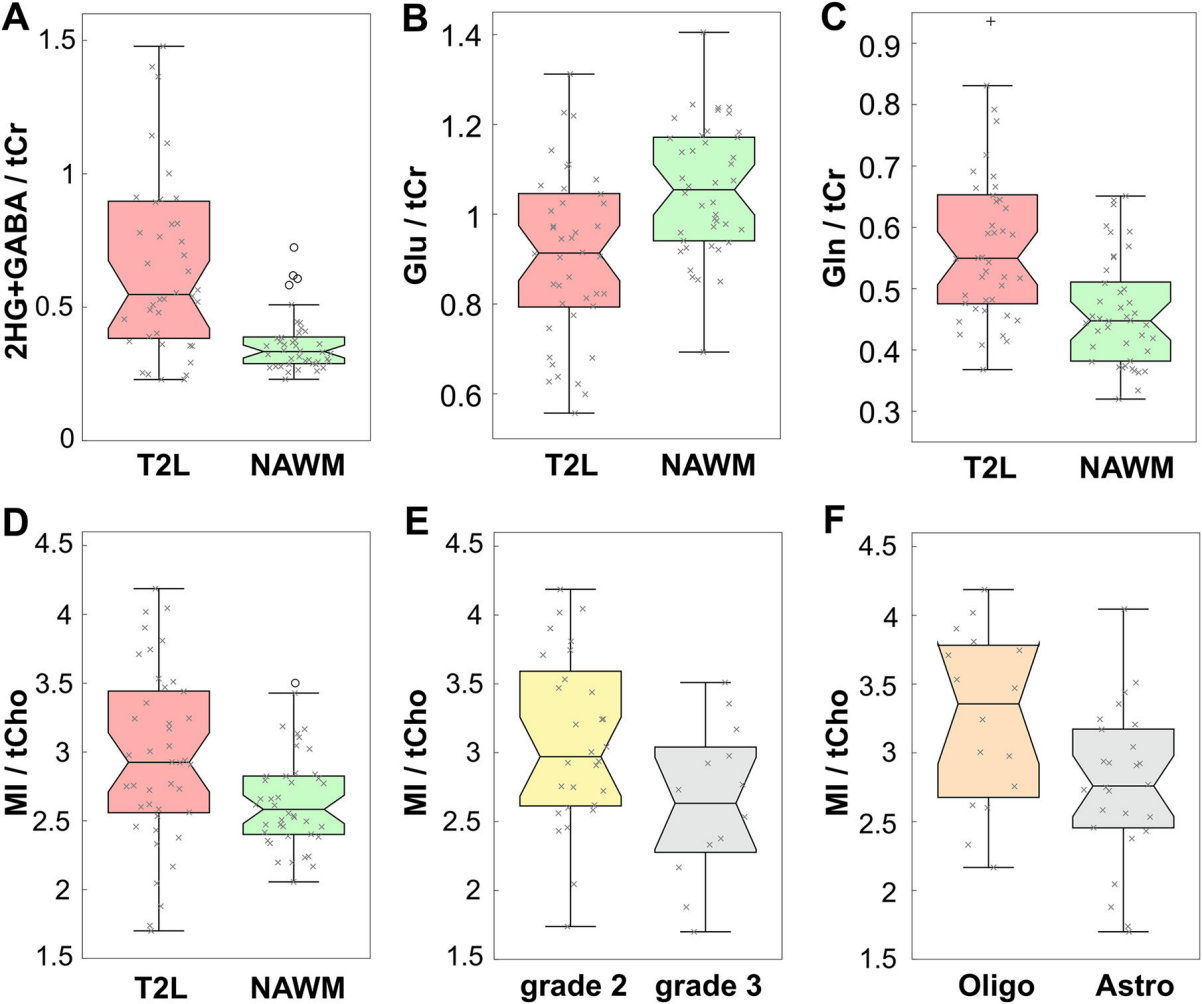
MRSI in IDH+ patients. 3D MRSI data quantified by LCModel showing the ratios of 2HG+ GABA/tCr (**A**), Glu/tCr (**B**), Gln/tCr (**C**) and MI/tCho (**D**) in patients with newly diagnosed IDH-mutant gliomas: T2L versus NAWM. The ratio of MI/tCho is also shown for the comparison of grade 2 versus 3 (**E**) and oligodendroglioma versus astrocytoma (**F**). See Table 2

MRSI spectra from T2Ls displayed apparent quantifiable levels of 2HG in 39/42(93%) IDH+ and 1/3(33%) IDH− patients, thus providing an overall detection accuracy of 91.1% for the entire LrGG population. Table 2 presents a summary of significant features describing newly diagnosed IDH+ lesions. Among newly diagnosed IDH+ patients (*n* = 42), levels of 2HG + GABA/tCr in the T2L were significantly elevated compared to NAWM (*p* < 1.0 × 10^−5^); whereas levels of Glu/tCr (*p* < 1.0 × 10^−3^) showed a corresponding reduction (Table 2; Fig. 2). Relative to NAWM, the IDH + T2L was also characterized by elevations in Gln/tCr (*p* = 0.001), MI/tCr (*p* < 1.0 × 10^−4^), MI + Gly (mIG)/tCr (*p* = 1.0 × 10*−9*), MI/tCho (*p* = 0.001), mIG/tCho (*p* < 1.0 × 10^−4^), tCho/tCr (*p* < 1.0 × 10^−5^) and tCho/tNAA (*p* < 1.0 × 10^−6^); and a reduction in tNAA/tCr (*p* < 1.0 × 10^−10^) (Table 2; Fig. 2).

Among newly diagnosed IDH+ LrGG, the grade 2 gliomas demonstrated an elevated ratio of MI/tCho (*p* = 0.020) compared to grade 3 counterparts (Table 2; Fig. 2). When evaluated according to histological subtype, oligodendrogliomas (81% grade 2; 19% grade 3) showed higher MI/tCho (*p* = 0.037) and mIG/tCho (*p* = 0.023) relative to astrocytomas (52% grade 2; 48% grade 3). Grade-based differences in histological subtypes were noted as a source of bias in this comparison. Additionally, astrocytic tumors, which were characterized by a greater proportion of grade 3 versus 2 pathology, consistently demonstrated the largest volumes of CNI_T2L_ > 2 (*p* < 0.01) and T2L (*p* < 0.05) (Table 2; Fig. 2).

Available p53 expression data among newly diagnosed IDH+ LrGG [18(69%) astrocytoma; 8(31%) oligodendroglioma] showed higher levels of tCho/tCr (*p* = 0.017), together with lower levels of MI/tCho (*p* = 0.021) and mIG/tCho (*p* = 0.018), when compared against p53-wildtype tumor [2(22%) astrocytoma; 7(78%) oligodendroglioma] (Table 2; Fig. 2). The p53-mutant tumors also presented larger volumes of CNI_T2L_ > 2 (*p* = 0.008) and T2L (*p* = 0.009) relative to non-mutant counterparts (Table 2).

### Spectroscopic and pathologic correlations

**Fig. 3:**
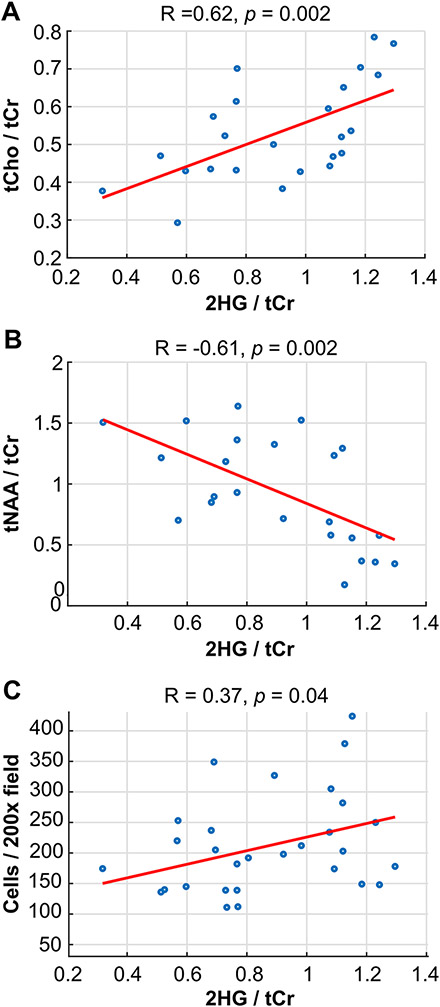
Image-guided tissue relationships. Correlations between levels of 2HG in patients with LrGG at the site of tissue sampling and tCho/tCr (**A**), tNAA/tCr (**B**) and pathological assessment of cellularity (**C**)

Tumor was verified by histopathology in 75/108 image-guided samples, 28 of which displayed reconstructed 2HG spectra at the site of sampling with CRLB < 25 (17 patients). Figure 3 shows 2HG levels in sampled regions correlated with tCho/tCr (*R* = 0.62, *p* = 0.002), tNAA/tCr (R = − 0.61, *p* = 0.002), and cellularity (R = 0.37, *p* = 0.04; CRLB < 30, *n* = 32); no correlation between 2HG and MIB-1 was observed. Evaluation of metabolite-pathology correlations across all tumor-containing samples, revealed tNAA was inversely correlated with cellularity (R = − 0.35, *p* = 0.002; *n* = 75); and CNI was correlated with MIB-1 (R = 0.27, *p* = 0.041, *n* = 58). MRSI metabolite correlations within the T2Ls of newly diagnosed IDH+ glioma are presented in Supplementary Fig. 1. CNI-related parameters were inversely correlated with levels of tNAA/tCr and MI/tCho; 2HG and tCho/tCr were weakly correlated.

### PFS analysis

The median PFS among all IDH+ patients was 3.8 years (95% CI 2.6–4.9 years; 17 censored), and 4.0 years (95% CI 2.7–5.3 years) in the newly diagnosed cohort (Supplementary Fig. 2A; 15 censored). Tumor grade, histological subtype and recurrence status were not significantly correlated with PFS. Cox proportional hazard models indicated that higher levels of 2HG/tCr (*p* = 0.0007, HR 5.594) and Glu/tCr (*p* = 0.006, HR 32.567), along with larger volumes of T2L (*p* = 0.02, HR 1.014) and non-enhancing lesion (*p* = 0.03, HR 1.014), were predictive of worse PFS in newly diagnosed patients (Supplementary Table 1). Higher levels of 2HG/tCr (≥ 0.905, median value; *p* = 0.02; Fig. S2B) and higher levels of Glu/tCr (≥ 0.945, median value; log-rank test, *p* = 0.02; Fig. S2C) were associated with a significant reduction in PFS (Table S1). Based on Cox regression analysis, there was no significant difference between populations empirically thresholded by 2HG/tCr or Glu/tCr levels with respect to extent of resection or treatment post surgery (Supplementary Tables 2 and 3).

## Discussion

This MRSI study demonstrated the detection and quantification of 2HG in patients with LrGG, who underwent image-guided tissue sampling during surgery to assess regional histopathology. Among IDH+ LrGG patients, the full complement of metabolite data derived from 3-D MRSI helped to noninvasively differentiate tumor with respect to pathological grade, histological subtype (1p/19q codeletion), p53 expression, and NAWM. Additionally, levels of 2HG quantified from data that was reconstructed at tissue sampling locations correlated with other metabolites and cellularity. Based on empirical thresholding of lesion-wide 2HG levels, it was possible to distinguish a subpopulation of IDH+ patients that manifested adverse PFS associated with elevations of the oncometabolite.

Using a clinically feasible MRS strategy for detecting 2HG with enhanced 3-D coverage and 1-cm^3^ resolution (*t*_acq_ = 8.2 min.), this study achieved 91% apparent accuracy in predicting IDH mutational status, which is in line with prior literature detailing technical reliability [16, 17]. However, this assessment of diagnostic accuracy must be contextualized by the considerable class imbalance created by the 3 IDH− patients relative to the larger overall population and the absence of a validation cohort to enrich the number of IDH− patients.

While a majority (93%) of patients expressing mutant IDH displayed 2HG-positive quantification, 1/3(33%) patients who lacked immunohistochemical and sequencing evidence of IDH mutation nevertheless indicated 2HG presence. This discordant case was evaluated by IHC for the most common IDH1^R132H^-mutant enzyme, which accounts for approximately 90% of identified IDH1 mutations [1], and also sequenced for the range of IDH1 R132/R100 and rarer IDH2 R172/R140 mutational variants [31]. As a matter of technical limitation, such false identification of 2HG can arise with LCModel quantification of background metabolites GABA, Glu and Gln, as well as lipids from necrotic lesions [32, 33], resonating in the H4,H4’-proton region of 2HG around 2.26 ppm. Other challenges to absolute detection can stem from partial volume effects in small lesions [20] and relatedly low 2HG concentrations (< 1 mM) [18] (here indicated by relatively higher tNAA levels), coupled with the relatively high spectroscopic spatial resolution that limited 2HG signal most noticeably from data reconstructed at tissue sampling sites (28/75).

Because the study population was overwhelmingly comprised of patients with IDH+ LrGG, it was difficult to assess the broader metabolic implications of IDH mutations [10] relative to IDH-lesions. Earlier investigations have demonstrated glutamate reduction in IDH-mutagenized cell lines [34], surgically-derived IDH+ tumor samples [35, 36], and high-grade IDH+ lesions (7 T MRS) [37] compared to non-mutated counterparts, perhaps as a consequence of conversion to 2HG-precursor alpha-ketoglutarate via glutamate dehydrogenase [36, 38]. Some of the same studies additionally reported increased glutamine levels in IDH+ versus IDH− lesions, which may represent heightened metabolic dependency in the mutated phenotype [34, 36, 37]. Although primarily treating IDH+ lesions here, the characteristically reduced glutamate and elevated glutamine levels in tumor versus NAWM follow similar trends.

Besides 2HG production and apparent alterations to glutamine/glutamate metabolism, the IDH+ T2Ls displayed a variety of features distinguishing them from NAWM. The elevation in choline species, together with reduced levels of tNAA, was consistent with established literature characterizing increased cell turnover and reduced neuronal function in glioma, respectively [39, 40]. Several studies have suggested that increased MI within the T2L versus NAWM can be understood as the result of heightened glial cellularity, particularly in astrocytomas [41]. As a known marker of malignant tumor, the elevation in Gly, which was fit in combination with MI due to spectral overlap, represents an expected finding [42]. The ratio of MI/tCho, previously evaluated as a marker of gliosis versus recurrent tumor [43], was shown to be reduced in the lesion owing to the excess choline content.

Despite lacking practical utility in discerning tumor lesions from NAWM, the ratio of MI/tCho was shown to function as a broad indicator of inter-tumoral malignancy when performing pairwise comparisons of molecular/pathological glioma classifications. A major caveat to this finding, however, was that aggressive tumors, which demonstrated an inverse relationship with MI/tCho, tended to be astrocytic (1p/19q-wildtype) and of higher grade. Higher values of MI/tCho, for example, provided the best means of discriminating grade 2 versus 3 IDH+ glioma, with prior research showing effective stratification of grades 2–4 [44]. This ratio was likewise found to be higher in the least aggressive forms of IDH+ glioma across histological and molecular sub-types: e.g., oligodendroglial (1p/19q co-deleted) and p53-wildtype. Volumetric trends showing smaller T2L and CNI_T2L_ > 2 regions in p53-wildtype and oligodendroglial tumors also distinguished less aggressive gliomas.

2HG correlations within surgically sampled regions supported a conventional understanding of the tumor microenvironment. While showing threshold-level significance, the relationship observed between 2HG and cellularity comports with the notion that tumor cell density influences 2HG productive capacity, as evidenced by other pre-surgical studies [12, 13, 18, 20] and those employing ADC as a surrogate for cellularity [20, 45]. Image-guided samples of tumors displayed a correlation between pathological measurement of cellular proliferation via MIB-1 and the spectroscopic marker of CNI, consistent with prior research [46]. Although 2HG was not found to be associated with the MIB-1 index, metabolite correlations with 2HG highlighted the abovementioned elevation of total choline and concomitant reduction in tNAA, which have historically been used as non-invasive measures of tumor malignancy. Despite the challenges that image-guided studies pose in terms of tissue shift and image-tissue mis-registration, the technical implementation of 3-D MRSI coverage demonstrated in this study increased the opportunity for evaluating in vivo correlates of sampled tissue.

Of particular importance in this study were the findings that adverse PFS could most reliably be predicted by increasing levels of 2HG and glutamate, the latter having previously been associated with malignant progression [35]. These results suggest that spectroscopic measures of 2HG and other metabolites provide information that extends beyond the mere identification of IDH mutation by IHC and genomic analyses. Moreover, empirically thresholding levels of 2HG in IDH+ patients enabled a practical means of discriminating subpopulations with disparate PFS, showing the potential utility of 2HG as a prognostic marker. With the current challenges in monitoring response to treatment across a range of standard-of-care and experimental therapies, several promising studies have already sought to leverage this utility by capturing longitudinal changes in 2HG associated with disease progression and radiochemotherapy using MRS [18-20]. The collective advances being made in 2HG imaging lend support to the emergent clinical value of in vivo spectroscopy and its potential to enhance existing MR protocols for patients with LrGG.

## Conclusion

In vivo 2HG detection can be achieved on clinical scanners and may have prognostic value based on the relationship between quantified levels and PFS. Spectroscopic strategies for resolving 2HG benefit from the technical implementation of 3-D MRSI that extends volumetric coverage.

## Supplementary Material

Supplementary tables

Supplementary Fig1

Supplementary Fig2

## Figures and Tables

**Table 1:** Patient profile. Clinical, molecular, and spectroscopic characteristics of patients with new and recurrent LrGG

Tumor classification	Patients (%)
LrGG (*n* = 45)	
Male/Female	27/18 (60/40%)
Grade 2/3	30/15 (67/33%)
Newly diagnosed	40 (89%)
Recurrent	5 (11%)
IDH+ glioma (*n* = 42)	
Grade 2/3	29/13 (69/31%)
Oligodendroglioma	16 (38%)
Astrocytoma	26 (62%)
p53+ (*n* = 31/37)	
2HG quantifiable	39 (93%)
IDH− glioma (*n* = 3)	
Grade 2/3	1/2 (33/67%)
Oligodendroglioma	0 (0%)
Astrocytoma	3 (100%)
p53+ (*n* = 1/2)	
2HG quantifiable	1 (33%)

**Table 2:** Features of newly diagnosed IDH + LrGG. Statistically significant MRSI and volumetric parameters among newly diagnosed patients with IDH+ LrGG; 2HG/tCr was reported regardless of significance for each categorical comparison

LrGG comparison	Parameter	Patients (*n*_1_, *n*_2_)	Values median (lower, upper quartile)		*p*–value
T2L vs NAWM			T2L	NAWM	
	2HG+ GABA/tCr	36, 36	0.54 (0.38,0.83)	0.35 (0.30,0.39)	< 0.001
	Glu/tCr	37, 37	0.95 (0.81,1.06)	1.06 (0.96,1.18)	< 0.001
	Gln/tCr	37, 37	0.54 (0.47,0.64)	0.45 (0.40,0.51)	< 0.001
	MI/tCho	37, 37	2.94 (2.60,3.47)	2.55 (2.40,2.82)	0.001
	MI/tCr	37, 37	1.44 (1.21,1.69)	1.03 (0.94,1.09)	< 0.001
	mIG/tCr	37, 37	1.64 (1.33,1.87)	1.08 (0.99,1.20)	< 0.001
	mIG/tCho	37, 37	3.31 (2.92,3.75)	2.75 (2.57,3.14)	< 0.001
	tCho/tNAA	37, 37	0.35 (0.29,0.47)	0.20 (0.18,0.21)	< 0.001
	tCho/tCr	37, 37	0.47 (0.43,0.53)	0.39 (0.36,0.43)	< 0.001
	tNAA/tCr	37, 37	1.32 (1.02–1.51)	1.97 (1.88,2.13)	< 0.001
Grade 2 vs 3			Grade 2	Grade 3	
	MI/tCho	24, 13	3.12 (2.75,3.72)	2.73 (2.33,2.98)	< 0.05
	2HG/tCr	22, 12	0.90 (0.76,1.07)	0.92 (0.67,1.17)	NS
Astro vs Oligo			Astro	Oligo	
	MI/tCho	21, 16	2.91 (2.53,3.17)	3.36 (2.72,3.76)	< 0.05
	mIG/tCho	21, 16	3.16 (2.75,3.46)	3.68 (3.22,4.06)	< 0.05
	2HG/tCr	19, 15	0.90 (0.65,1.00)	0.93 (0.81,1.15)	NS
	Vol. CNI_T2L_ > 2 (cm^3^)	21, 16	19.9 (16.2,38.0)	10.0 (6.2,16.8)	< 0.01
	Vol. T2L, grade 2 (cm^3^)	11, 13	63.6 (30.1,75.0)	20.4 (13.9,34.4)	< 0.05
p53+ vs p53−			p53+	p53−	
	tCho/tCr	26, 7	0.49 (0.44,0.56)	0.42 (0.38,0.45)	< 0.05
	MI/tCho	26, 7	2.83 (2.42,3.23)	3.71 (3.08,3.96)	< 0.05
	mIG/tCho	26, 7	3.14 (2.75,3.64)	4.00 (3.48,4.39)	< 0.05
	2HG/tCr	23, 7	0.93 (0.81,1.15)	0.81 (0.78,0.87)	NS
	Vol. CNI_T2L_ > 2 (cm^3^)	26, 7	18.5 (13.9,37.5)	7.8 (6.5,10.7)	< 0.01
	Vol. T2L (cm^3^)	26, 7	46.5 (30.6,74.9)	19.2 (12.3,33.4)	< 0.01

*2HG*, 2-hydroxyglutarate; *GABA*, γ-aminobutyric acid; *tCr*, creatine + phosphocreatine; *Glu*, glutamate; *MI*, myo-inositol; *tCho*, phosphocholine + glycerophosphocholine + choline; *Gly*, glycine; *tNAA*, *N*-acetylaspartate + *N*-acetylaspartylglutamate; *mIG*, MI + Gly; *CNI*, choline-to-NAA index; *Vol*, volume; *NS*, not significant

## Data Availability

Data available upon request.
