# Supplementary material for: Spectroscopic imaging of D-2-hydroxyglutarate and other metabolites in pre-surgical patients with IDH-mutant lower-grade gliomas
Source: J Neurooncol. Author manuscript; Available in PMC 2022 Aug 1. (PMC9325821; doi:10.1007/s11060-022-04042-3)
Supplement: Supplementary tables [file NIHMS1820622-supplement-Supplementary_tables.docx]

**Supplementary Table 1**. **Assessing** **PFS**. Parameters predicting adverse PFS in patients with newly diagnosed LrGG are displayed in order of relative hazard, together with empirically-thresholded classifiers that predicted worse PFS. NEL, non-enhancing lesion.

| **Predictor** | **Hazard ratio** | ***p*-value** |
| --- | --- | --- |
| Glu / tCr | 32.567 | 0.0006 |
| 2HG / tCr | 5.594 | 0.0007 |
| Vol. T2L | 1.014 | 0.02 |
| Vol. NEL | 1.014 | 0.03 |
| **Empirical Classifier** | **Outcome** | ***p*-value** |
| 2HG / tCr ≥ 0.905 | Worse PFS | 0.02 |
| Glu / tCr ≥ 0.945 | Worse PFS | 0.02 |

**Supplementary Table 2**. **Treatment in 2HG-thresholded PFS populations**. Extent of resection and post-surgical treatment in both patient populations empirically thresholded by levels of 2HG / tCr, which displayed disparate PFS. Treatment was similar across the two populations. GTR, gross total resection; STR, subtotal resection; RT, radiation therapy; TMZ, temozolomide.

|  | **Treatment** | **Patient Population** | | **Total** |
| --- | --- | --- | --- | --- |
|  |  | **2HG/tCr <0.905** | **2HG/tCr ≥0.905** |  |
| **Extent of Resection** | GTR | 7 | 8 | 15 |
|  | STR | 13 | 9 | 22 |
|  | **Total** | 20 | 17 | 37 |
| **Post-Surgical Treatment** | Everolimus | 2 | 1 | 3 |
|  | RT+TMZ | 7 | 5 | 12 |
|  | TMZ | 1 | 3 | 4 |
|  | No Treatment | 9 | 8 | 17 |
|  | Unknown (Lost Visit) | 1 | 0 | 1 |
|  | **Total** | 20 | 17 | 37 |

**Supplementary Table 3**. **Treatment in Glu-thresholded PFS populations**. Extent of resection and post-surgical treatment in both patient populations empirically thresholded by levels of Glu / tCr, which displayed disparate PFS. Treatment was similar across the two populations. GTR, gross total resection; STR, subtotal resection; RT, radiation therapy; TMZ, temozolomide.

|  | **Treatment** | **Patient Population** | | **Total** |
| --- | --- | --- | --- | --- |
|  |  | **Glu/tCr <0.945** | **Glu/tCr ≥0.945** |  |
| **Extent of Resection** | GTR | 7 | 8 | 15 |
|  | STR | 11 | 11 | 22 |
|  | **Total** | 18 | 19 | 37 |
| **Post-Surgical Treatment** | Everolimus | 1 | 2 | 3 |
|  | RT+TMZ | 7 | 5 | 12 |
|  | TMZ | 2 | 2 | 4 |
|  | No Treatment | 8 | 9 | 17 |
|  | Unknown (Lost Visit) | 0 | 1 | 1 |
|  | **Total** | 18 | 19 | 37 |
