# Supplementary figures and images for: Spectroscopic imaging of D-2-hydroxyglutarate and other metabolites in pre-surgical patients with IDH-mutant lower-grade gliomas
Source: J Neurooncol. Author manuscript; Available in PMC 2022 Aug 1. (PMC9325821; doi:10.1007/s11060-022-04042-3)

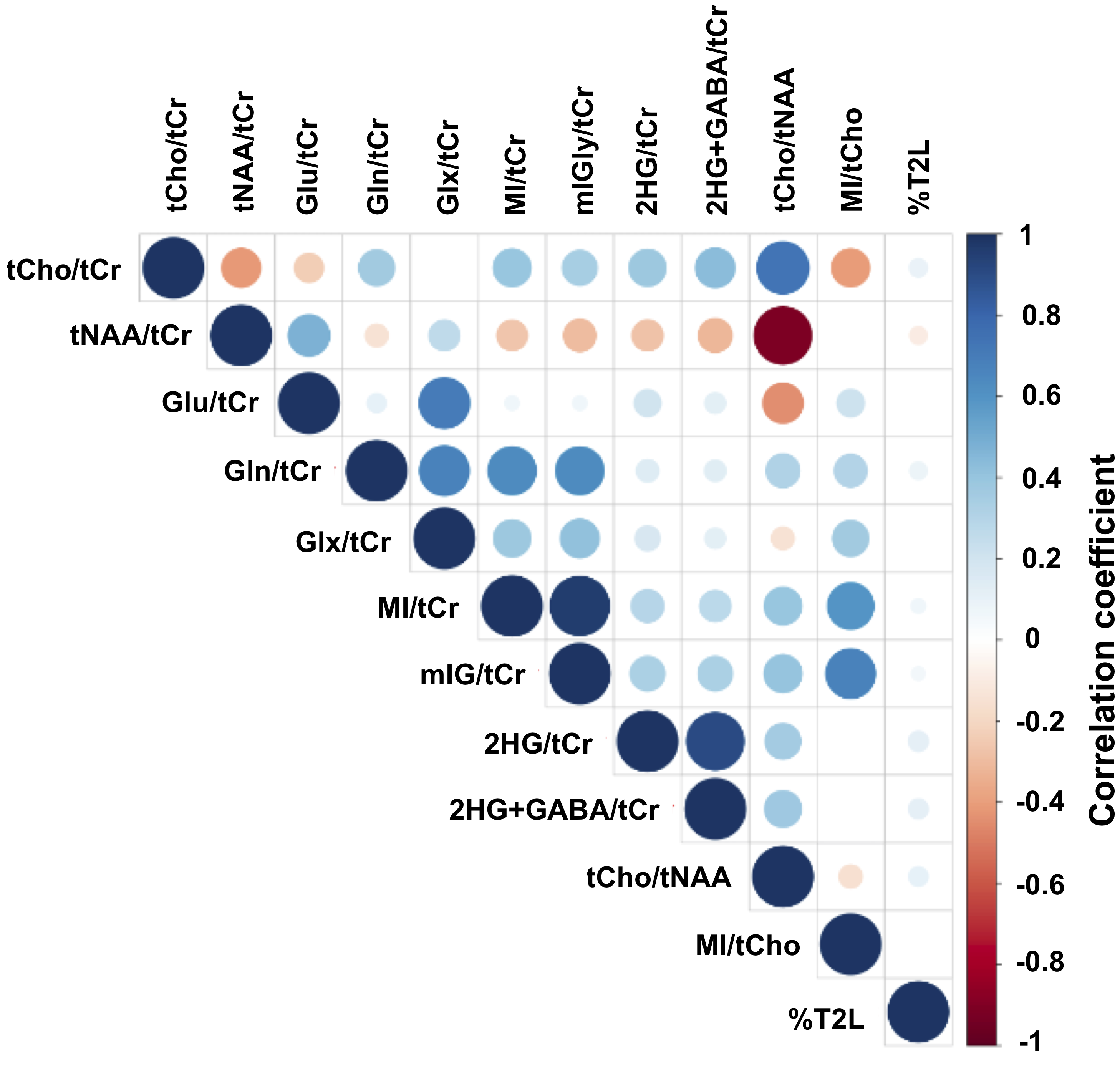

Supplement: Supplementary Fig1 [file NIHMS1820622-supplement-Supplementary_Fig1.tiff]

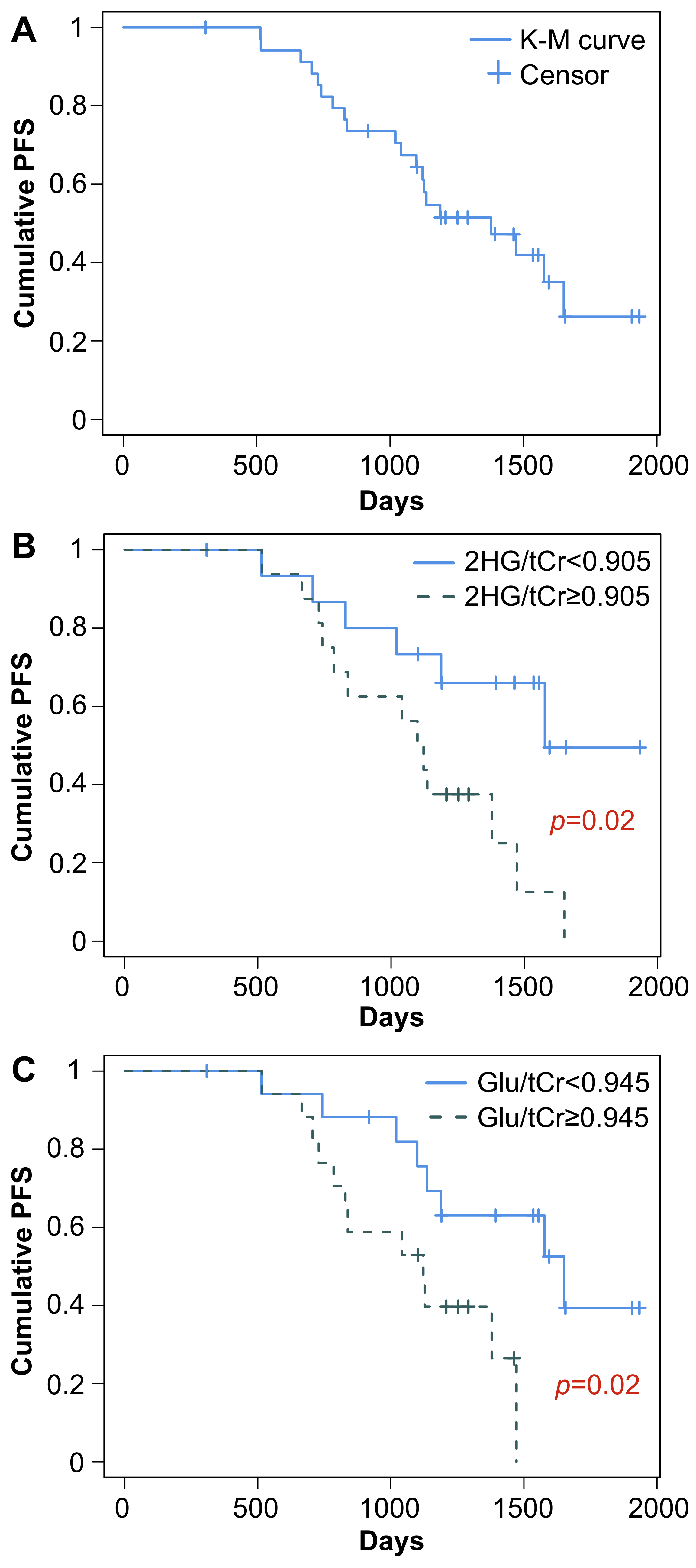

Supplement: Supplementary Fig2 [file NIHMS1820622-supplement-Supplementary_Fig2.tiff]
